# Rho/ROCK Inhibition Promotes TGF-*β*3-Induced Tenogenic Differentiation in Mesenchymal Stromal Cells

**DOI:** 10.1155/2021/8284690

**Published:** 2021-10-08

**Authors:** Michaela Melzer, Susanna Schubert, Simon Franz Müller, Joachim Geyer, Alina Hagen, Sabine Niebert, Janina Burk

**Affiliations:** ^1^Equine Clinic (Surgery, Orthopedics), Faculty of Veterinary Medicine, Justus-Liebig-University Giessen, 35392 Giessen, Germany; ^2^Saxon Incubator for Clinical Translation, University of Leipzig, 04103 Leipzig, Germany; ^3^Institute of Human Genetics, University of Leipzig Hospitals and Clinics, 04103 Leipzig, Germany; ^4^Biomedical Research Center Seltersberg (BFS), Institute of Pharmacology and Toxicology, Faculty of Veterinary Medicine, Justus-Liebig-University Giessen, 35392 Giessen, Germany

## Abstract

Mesenchymal stromal cells (MSC) represent a promising therapeutic tool for tendon regeneration. Their tenogenic differentiation is crucial for tissue engineering approaches and may support their beneficial effects after cell transplantation *in vivo*. The transforming growth factor (TGF)-*β*, signalling via intracellular Smad molecules, is a potent paracrine mediator of tenogenic induction. Moreover, scaffold topography or tendon matrix components induced tenogenesis via activation of the Rho/ROCK cascade, which, however, is also involved in pathological adaptations in extracellular matrix pathologies. The aim of this study was to investigate the interplay of Rho/ROCK and TGF-*β*3/Smad signalling in tenogenic differentiation in both human and equine MSC. Primary equine and human MSC isolated from adipose tissue were cultured as monolayers or on tendon-derived decellularized scaffolds to evaluate the influence of the ROCK inhibitor Y-27632 on TGF-*β*3-induced tenogenic differentiation. The MSC were incubated with and without TGF-*β*3 (10 ng/ml), Y-27632 (10 *μ*M), or both. On day 1 and day 3, the signalling pathway of TGF-*β* and the actin cytoskeleton were visualized by Smad 2/3 and phalloidin staining, and gene expression of signalling molecules and tendon markers was assessed. ROCK inhibition was confirmed by disruption of the actin cytoskeleton. Activation of Smad 2/3 with nuclear translocation was evident upon TGF-*β*3 stimulation. Interestingly, this effect was most pronounced with additional ROCK inhibition in both species (*p* < 0.05 in equine MSC). In line with that, the tendon marker scleraxis showed the strongest upregulation when TGF-*β*3 and ROCK inhibition were combined (*p* < 0.05 in human MSC). The regulation pattern of tendon extracellular matrix components and the signalling molecules TGF-*β*3 and Smad 8 showed differences between human and equine MSC. The obtained results showed that ROCK inhibition promotes the TGF-*β*3/Smad 2/3 axis, with possible implications for future MSC priming regimes in tendon therapy.

## 1. Introduction

Tendon injuries are frequently occurring diseases in horses and humans [1–4]. Due to their hypocellular, hypovascular nature, tendons have only an insufficient spontaneous regeneration capability [5, 6]. As a result, conventional therapies are usually very protracted and associated with a high rate of reinjury. Therefore, tissue engineering and cell therapies are promising options. Several equine studies have already found evidence for positive effects of multipotent mesenchymal stromal cells (MSC) on tendon healing [7–9], but the full potential of this approach has yet to be understood and exploited. Successful tenogenic differentiation of MSC is crucial for tissue engineering approaches and may play an important role with respect to long-term effects of MSC-based cell therapies, along with initial immunomodulation and support of M2 macrophages [10, 11]. However, tenogenic differentiation appears to depend on several external factors, and their interplay is still widely unclear.

Reported tenogenic factors include the extracellular tendon matrix (ECM) as well as growth factors, particularly transforming growth factor (TGF)-*β*. TGF-*β* is one of the most extensively researched growth factors and is well established as a reliable inducer for tenogenic differentiation [12–14]. The canonical signalling pathway of TGF-*β* involves downstream Smad molecules. TGF-*β* binding to the TGF-*β* family receptor, a serine/threonine kinase cell surface receptor, results in direct carboxy-terminal phosphorylation of Smad 2 and 3. These build a complex with co-Smads, translocate into the nucleus, and induce gene transcription together with other coregulators [15, 16].

Regarding the ECM, it has been shown that both its structure and biochemical components play an important role in tenogenic differentiation [17–19]. Not only did scaffolds with aligned fibers promote tenogenic differentiation but also they inhibited osteogenic induction compared to randomly oriented fiber scaffolds [17]. Furthermore, tendon-derived urea-extracted ECM solution exerted tenogenic effects on MSC [18]. The Rho/ROCK pathway, which is activated by integrins and responsible for cytoskeleton maintenance, was proposed to mediate mechanically induced tenogenic differentiation, as its inhibition using the ROCK inhibitor Y-27632 prevented tenogenic differentiation triggered by mechanical stimulation [20] or scaffold topography [21].

Based on their independently reported positive effects on tenogenic differentiation, synergistic effects of Rho/ROCK and TGF-*β*3-induced Smad 2/3 signalling might be expected, possibly depending on the microenvironment responsible for Rho/ROCK activation. Decellularized tendon matrix used as a scaffold favorably mimics the tendon microenvironment, as both the tendon ECM architecture and its biochemical composition are represented. However, in previous experiments with equine MSC, we observed an upregulation of the tendon marker scleraxis upon TGF-*β*3 stimulation only in monolayer cultures, but not in decellularized tendon scaffold cultures [22], warranting clarification of the underlying mechanisms. Furthermore, the implication of Rho/ROCK in fibrotic maladaptations [23] makes it crucial to further elucidate its role in tendon regeneration.

The aim of this study was to investigate the interplay between the Rho/ROCK pathway and Smad 2/3 activation by TGF-*β*3 in the context of early tenogenic differentiation, using human as well as equine MSC in monolayer and tendon scaffold cultures in the presence of TGF-*β*3 and/or the ROCK inhibitor Y-27632.

## 2. Materials and Methods

### 2.1. MSC Isolation and Culture

Human subcutaneous adipose tissue was obtained from 3 healthy human donors (age: 32–48 years; female) after liposuction for cosmetic reasons. All human samples were collected with informed consent of the donors and approval by the ethics committee of the Medical Faculty, University of Leipzig (096/17-ek). Equine subcutaneous adipose tissue was harvested from the supragluteal region from 3 healthy Standardbred horses (age: 5–9 years; male) within the framework of an unrelated previous study as approved by the respective local authority (Landesdirektion Leipzig, TV34/13). All donors were unrelated to each other.

Primary MSC were isolated by collagenase digestion (0.8 mg/ml collagenase type I) for 2–4 h at 37°C with permanent shaking. Isolated cells were then seeded and expanded in their respective standard culture medium and cryopreserved at passage 1 or 2. Human MSC were cultured in alpha Minimum Essential Medium (MEM Alpha Medium+GlutaMAX™, Gibco®), supplemented with 2.5% human platelet lysate (PL BioScience GmbH, Aachen, Germany), 1% penicillin-streptomycin, 0.1% gentamycin, and 1 U/ml heparin (PL BioScience GmbH). Equine MSC were cultured in low-glucose (1 g/l) Dulbecco's modified Eagle's medium (Gibco®), supplemented with 10% fetal bovine serum (Gibco®), 1% penicillin-streptomycin, and 0.1% gentamycin. Collagenase, media, and supplements were purchased from Thermo Fisher Scientific GmbH (Dreieich, Germany) unless stated otherwise.

All MSC were characterized by differentiation assays and flow cytometry as recommended by the International Society for Cell and Gene Therapy [24]. Briefly, differentiation was performed using the StemPro™ Differentiation Kits (Thermo Fisher Scientific GmbH) and evaluated qualitatively based on standard staining (Oil Red O, von Kossa, and Alcian blue for adipogenic, osteogenic, and chondrogenic differentiation, respectively). Chondrogenic differentiation was only evaluated in equine MSC due to limited human MSC cell numbers. Surface antigen staining and flow cytometry were performed as described previously for human [25] and equine [26] MSC. Additionally, the effect of the ROCK inhibitor Y-27632 on human and equine MSC was tested in preliminary experiments.

### 2.2. Scaffold Preparation

Equine tendon tissue was harvested from the superficial digital flexor tendon of limbs obtained at an abattoir. After storage at -80°C, tendons were decellularized with freeze-thaw cycles according to a previous published protocol [27] and stored in a sterile bag at –80°C until use. To adapt the scaffold size, tendons were first cut manually to a size of 10 mm × 10 mm, then placed in a cryostat CM 3050 S (Leica Microsystems AG, Wetzlar, Germany) with a working temperature of –20°C, and sliced to 300 *μ*m thick scaffolds.

### 2.3. Cell Culture Experiments

MSC from each individual donor at passage 2 or 3 were used to repeat all experiments (*n* = 3 biological replicates for each species). Monolayer cells were seeded with a density of 3,000 cells/cm^2^ on standard cell culture plates. Tendon scaffolds were placed in 24-well plates with an ultralow attachment surface (Corning® Costar®, Corning GmbH, Kaiserslautern, Germany) and prewarmed for 10 min at 37°C. Next, 300,000 cells/30 *μ*l/cm^2^ were distributed on each scaffold and allowed to attach for 6 h. Thereafter, 1 ml standard culture medium was added, and seeded scaffolds were incubated at 37°C in a 5% CO_2_ atmosphere. After 3 days of cultivation, medium was changed and monolayers as well as seeded scaffolds were stimulated with 10 ng/ml TGF-*β*3 (R&D Systems®, Minneapolis, MN, USA), 10 *μ*M Y-27632 (Tocris, Bioscience, Bristol, UK), or both. For the last one, cells were preincubated with Y-27632 for 2 h before adding TGF-*β*3. On days 1 and 3 after stimulation, tenogenic differentiation and the Smad 2/3 signalling pathway were assessed based on gene expression and fluorescence microscopy.

### 2.4. Real Time RT-PCR

Gene expression of the signalling molecules Smad 3, Smad 8, and TGF-*β*3, the tenogenic differentiation marker scleraxis, and the tendon extracellular matrix components collagen 1A2, collagen 3A1, and tenascin-C was analysed by real-time PCR. GAPDH was used as a reference gene. Total RNA of monolayer cells was isolated using the RNeasy Mini Kit (Qiagen, Hilden, Germany) with additional DNase digestion (Qiagen) according to instructions of manufacturers. Frozen MSC-seeded tendon scaffolds were homogenized with the Tissue Lyser II (Qiagen). After complete homogenization with proteinase k at 55°C (Qiagen), total RNA was isolated with the RNeasy Mini Kit. RNA was then converted to cDNA using the Reverse Transcriptase RevertAid H Minus (Thermo Fisher Scientific GmbH). 1 *μ*g cDNA was mixed with primers (Tables 1 and 2) and iQ SYBR Green Supermix (Bio-Rad Laboratories GmbH, Feldkirchen, Germany), and real-time PCR was performed using a 7500 Real-Time PCR System (Applied Biosystems™, Thermo Fisher Scientific) for the human samples and a qTower^3^G (Analytik Jena GmbH, Jena, Germany) for the equine samples. For relative quantification, gene expression ratios were calculated with the Pfaffl method, using additional monolayer day 0 samples as controls for normalization.

### 2.5. Immunofluorescence Staining

To visualize the effect of Y-27632 on the actin cytoskeleton as well as potential translocation of Smad 2/3 to the nucleus, the monolayer groups were stained with phalloidin and an anti-Smad 2/3 antibody. First, cells were fixed with 4% formaldehyde (Carl Roth® GmbH, Karlsruhe, Germany) for 10 min, and blocking solution (PBS+1% BSA (Carl Roth®)+0.3% Triton-X100 (Carl Roth®)+10% goat serum (Sigma-Aldrich GmbH, München, Germany)) was added for 1 h at room temperature. Next, the primary antibody (anti-Smad 2/3, 5678S, Cell Signaling Technology, Danvers, MA, USA) was diluted to 1 : 125 in PBS+1% BSA+0.3% Triton-X100 and incubated overnight at 4°C. A secondary anti rabbit antibody (Alexa Fluor 488 F(ab)` fragment of anti-rabbit antibody, 1 : 1000, Thermo Fisher Scientific GmbH) was then added for 1 h protected from light. Finally, cells were incubated with phalloidin (Dylight 554, 13054S, 1 : 200, Cell Signaling Technology) and DAPI (1 : 1000, Carl Roth®) for 20 min. Between each step, cells were washed with PBS three times for 5 min.

### 2.6. Microscopy and Image Analyses

Directly after staining, two images were obtained from each replicate at standardized settings using a Keyence HS All-In-One Fluorescence Microscope (Keyence, Osaka, Japan) for human cells and a Leica DMI6000 B (Leica Microsystems) for equine cells. For image quantification, FIJI ImageJ was used. Two images per replicate were used for quantification. First, an individual background subtraction was performed for each image. Then, cells were segmented into the cytoplasm and nucleus based on DAPI and phalloidin staining to create region of interest (ROI) masks. The ROI masks were used to measure mean intensity values of Smad 2/3 staining in the whole cell, as well as in the nuclei and cytoplasm separately. Total Smad 2/3 staining in the whole cell was normalized to DAPI staining to investigate the amount of Smad 2/3 present in each cell, and the Smad 2/3 nucleus to cytoplasm ratio was calculated to quantify nuclear translocation of the molecule.

### 2.7. Statistics

Statistical analysis was performed using IBM SPSS Statistics 26 software (IBM Deutschland GmbH, Ehningen, Germany). Biological replicates were considered separate cases, while data from technical repetitions were averaged before further analysis. As data were not normally distributed, nonparametric Friedman tests with Bonferroni-adjusted post hoc tests were used. Differences were considered significant at *p* < 0.05.

## 3. Results

### 3.1. MSC Characterization

MSC were capable of differentiation into adipocytes, osteoblasts, and chondrocytes. Human MSC were positive for CD29 (97.8–99.8%), CD73 (22.6–98.3%), CD90 (90.2–98.9%), and CD105 (46.7–97.7%) and widely negative for CD14 (0.3–1.1%), CD34 (2.1–5.4%), CD45 (0.6–4.7%), CD79*α* (0.1–2.1%), and MHCII (0.0–0.2%). Representative images and histograms are shown in Supplementary file 1. Equine MSC were positive for CD29 (40.2–55.5%), CD44 (83.0–93.1%), CD90 (64.7–84.4%), and CD14 (62.3–86.7%) and largely negative for CD34 (0.6–3.0%), CD45 (0.4–1.3%), CD79*α* (5.6–11.7%), MHCII (0.3–1.1%), CD73 (0.41–1.4%), and CD105 (0.1–1.0%). Representative images and histograms are shown in a previous publication [26]. When testing the ROCK inhibitor Y-27632, a concentration of 10 *μ*M was effective in disrupting the actin cytoskeleton and had no adverse effect on MSC viability, although a slight effect on proliferation was observed until day 3 (*p* > 0.05).

### 3.2. MSC Signalling

**Figure 1:**
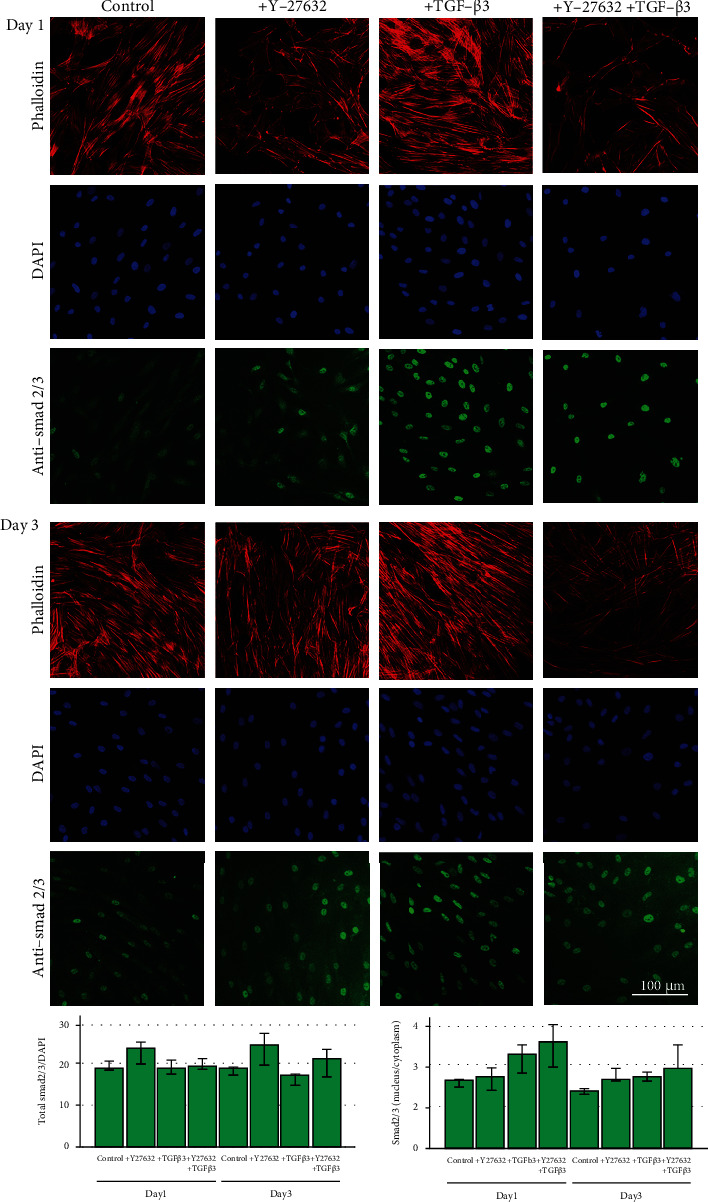
Fluorescence microscopy in human monolayer MSC. Cells were incubated with 10 ng/ml TGF-*β*3 and/or 10 *μ*M ROCK inhibitor Y-27632 and analysed at days 1 and 3. Representative images show phalloidin staining of the actin cytoskeleton, which is disrupted upon ROCK inhibition, nuclear staining with DAPI, and Smad 2/3 immunostaining with nuclear translocation upon TGF-*β*3 and TGF-*β*3 plus Y-27632 stimulation. Differences between groups are more clearly visible on day 1. The graphs represent the data from quantitative image analyses in cells from 3 donors, indicating a relatively constant amount of total Smad 2/3 (left) and nuclear translocation upon incubation with TGF-*β*3 and Y-27632 (right). Bars represent the median values and error bars the 95% confidence intervals.

**Figure 2:**
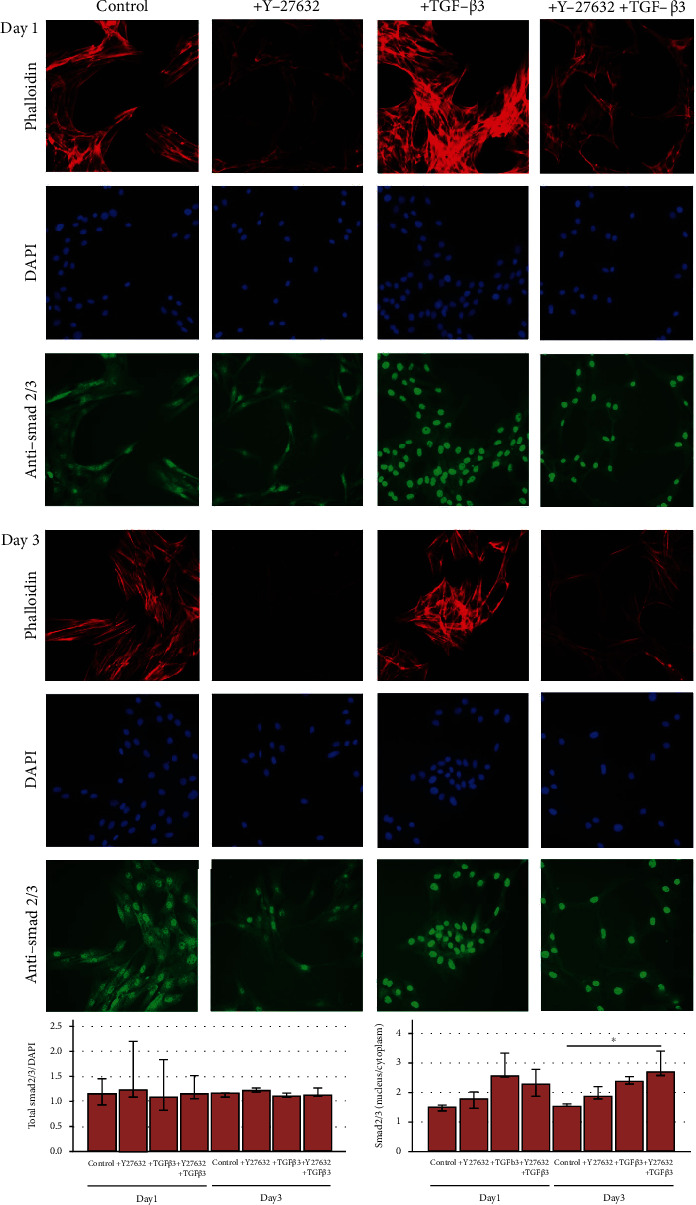
Fluorescence microscopy in equine monolayer MSC. Cells were incubated with 10 ng/ml TGF-*β*3 and/or 10 *μ*M ROCK inhibitor Y-27632 and analysed at days 1 and 3. Representative images show phalloidin staining of the actin cytoskeleton, which is disrupted upon ROCK inhibition, nuclear staining with DAPI, and Smad 2/3 immunostaining with nuclear translocation upon TGF-*β*3 and TGF-*β*3 plus Y-27632 stimulation. Differences between groups are clearly visible on days 1 and 3. The graphs represent the data from quantitative image analyses in cells from 3 donors, indicating a constant amount of total Smad 2/3 (left) and nuclear translocation upon incubation with TGF-*β*3 and Y-27632 (right). Bars represent the median values, error bars represent the 95% confidence intervals, and the asterisk marks a difference between the indicated groups with *p* < 0.05.

Inhibition of the Rho/ROCK signalling pathway with Y-27632 was confirmed by a loss of actin fiber organization, resulting in reduced fine drawing in phalloidin staining (Figures 1 and 2).

As expected, stimulation with TGF-*β*3 led to nuclear translocation of the signalling molecule Smad 2/3. Furthermore, the highest nucleus to cytoplasm ratios were obtained with TGF-*β*3 combined with ROCK inhibition by Y-27632, in human MSC on both days and in equine MSC on day 3 (*p* < 0.05 compared to control for equine MSC) (Figures 1 and 2).

**Figure 3:**
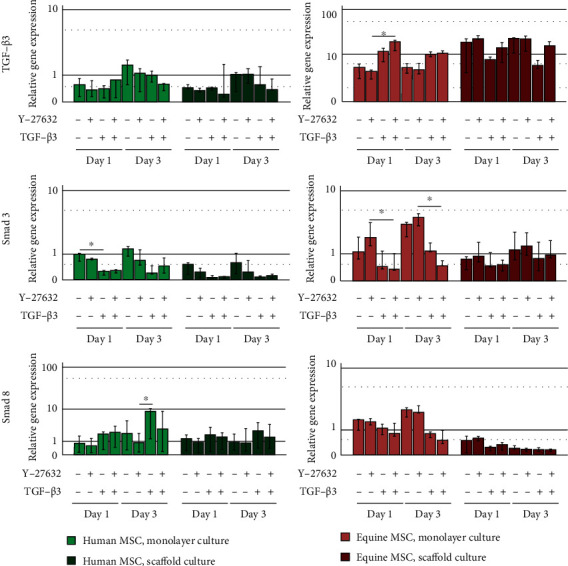
Gene expression of signalling molecules in human and equine MSC. Cells were cultured as monolayers or on decellularized tendon scaffolds and incubated with 10 ng/ml TGF-*β*3 and/or 10 *μ*M ROCK inhibitor Y-27632 and analysed at days 1 and 3. Bars represent the median values, error bars represent the 95% confidence intervals, and asterisks mark differences between the indicated groups with *p* < 0.05.

Gene expression of Smad 3 was downregulated in the presence of TGF-*β*3 or TGF-*β*3 and Y-27632 in both human and equine MSC. This trend was observed in monolayers as well as scaffold cultures, but differences were only significant for the monolayer cultures (*p* < 0.05 for human MSC, TGF-*β*3 vs. control group on day 3, and for equine MSC, TGF-*β*3 and Y-27632 group vs. Y-27632 group on days 1 and 3) and weak in equine scaffold cultures (Figure 3). However, Smad 3 downregulation was not reflected on protein level within the investigated time period, as total Smad 2/3 staining remained widely constant (Figures 1 and 2). Interestingly, while humans and horses showed a similar regulation of Smad 3, there were species differences in the regulation of Smad 8 and TGF-*β*3. In human MSC, Smad 8 expression was increased in the presence of TGF-*β*3, which was again more pronounced in monolayers (*p* < 0.05 for TGF-*β*3 vs. Y-27632 group on day 3), while equine MSC showed a contrary trend (Figure 3). TGF-*β*3 expression increased in the TGF-*β*3 group and in the TGF-*β*3 and Y-27632 group, but only in equine monolayer MSC (*p* < 0.05 for TGF-*β*3 and Y-27632 group vs. Y-27632 group on day 1). In equine scaffold cultures or human monolayer and scaffold cultures, this effect could not be confirmed or even tended to be reversed (Figure 3).

### 3.3. MSC Tenogenesis

**Figure 4:**
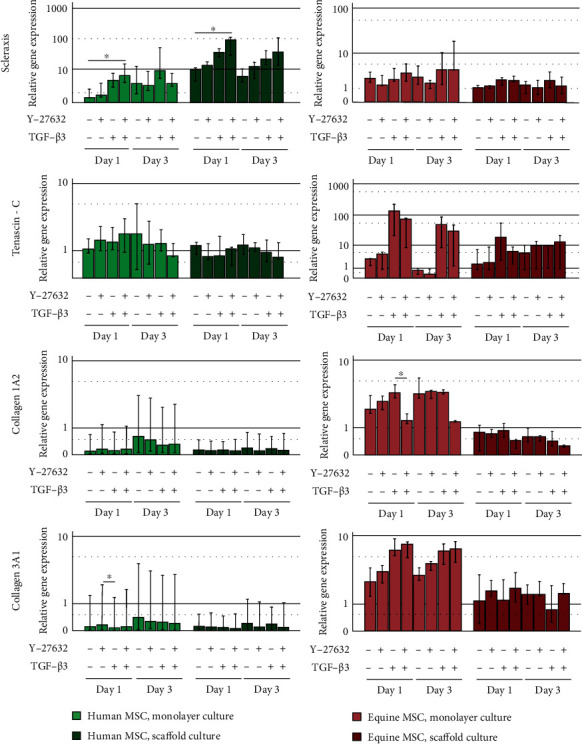
Gene expression of the tendon marker and extracellular matrix molecules in human and equine MSC. Cells were cultured as monolayers or on decellularized tendon scaffolds and incubated with 10 ng/ml TGF-*β*3 and/or 10 *μ*M ROCK inhibitor Y-27632 and analysed at days 1 and 3. Bars represent the median values, error bars represent the 95% confidence intervals, and asterisk mark differences between the indicated groups with *p* < 0.05.

The tendon marker scleraxis was upregulated in the presence of TGF-*β*3. This was reinforced with additional ROCK inhibition by Y-27632, which was particularly pronounced in human MSC (*p* < 0.05 compared to control in monolayer and scaffold cultures on day 1). The expression pattern of the extracellular matrix components differed between human and equine MSC. In human MSC, there were hardly any differences and high variability in the expression of tenascin-C and the collagens 1A2 and 3A1. Equine MSC showed an increased expression of tenascin-C in the TGF-*β*3 as well as in the TGF-*β*3 and Y-27632 groups, yet this was not repeatable in all donors and therefore not significant. Furthermore, the equine cells downregulated collagen 1A2 when TGF-*β*3 and Y-27632 were combined (*p* < 0.05 for TGF-*β*3 vs. TGF-*β*3 and Y-27632 group on day 1 in monolayers) but tended to upregulate collagen 3A1 (Figure 4).

## 4. Discussion

In this study, we investigated the crosstalk between the Rho/ROCK and TGF-*β*/Smad signalling pathways in the context of early tenogenic differentiation in MSC. Although synergistic effects of ROCK activity and TGF-*β*3 stimulation on tenogenesis might have been expected, we made the interesting observation that contrary to this assumption, inhibition of ROCK promoted TGF-*β*3-induced Smad 2/3 activity as well as tenogenic induction. This finding may have important implications for tissue engineering strategies and the local MSC transplantation in tendons.

The mechanisms of action of MSC are highly context-sensitive [28, 29], which include their adaptation to the local extracellular environment. This adaptation can enhance the cellular potency, as observed after targeted priming of MSC [30], but it could also compromise therapeutic mechanisms and success. In the context of musculoskeletal and connective tissue disease, possible MSC adaptations to the ECM could play a central role, particularly if the ECM is already pathologically altered being typical of tendon disease. In this context, Rho/ROCK signalling is of particular interest, as it is related to focal adhesion/integrin activation by the local ECM environment and thereby mediates mechanical stimuli from the extracellular space [31]. In this line, the Rho kinases have been identified as key players in fostering fibrotic disease [23]. Against this background, ECM and Rho/ROCK signalling may also play a role in the possible pathological adaptations of MSC to their environment.

With regard to tendon regeneration, however, Rho/ROCK signalling has so far been considered supportive, as it was described to be essential for tenogenic differentiation induced by scaffold topography [21] and mechanical stretching [20]. In these studies, ROCK inhibition resulted in decreased expression of tendon markers and less pronounced tenocyte-like morphology of MSC [20, 21]. Indicating a connection between mechanobiology, Rho/ROCK, and the well-described tenogenic effect of TGF-*β*, more recently, ECM extract-induced tenogenic differentiation was shown to be mediated via integrin as well as TGF-*β*/Smad pathways [32]. Furthermore, synergistic effects of ROCK activation by surface topography and TGF-*β*2 on Smad phosphorylation and tenogenic differentiation were reported [33], which is consistent with similar synergistic effects observed in contexts such as chondrogenic [34] or lung fibroblast differentiation [35]. Yet, none of these studies investigated the combined effect of actual ECM and TGF-*β*, and when combining decellularized tendon ECM scaffolds with TGF-*β*3 to promote tenogenic differentiation in equine MSC, there was no convincing evidence for a stringent synergistic tenogenic effect of the tendon ECM and TGF-*β*3 [22].

Therefore, we aimed to further investigate the role of ROCK in the context of TGF-*β*3-induced tenogenic differentiation, including experiments in tendon scaffold culture. The scaffold chosen for this study has been established, characterized, and used in several previous studies [19, 22, 26, 28, 36, 37]. As it consists of native decellularized tendon tissue, it combines the two most important influencing factors of the ECM, topography and matrix components, and represents the closest possible approximation to the environment of native tendon tissue. Furthermore, we included two clinically relevant species, humans and horses, to detect potential species-related differences. The central finding of the current study is that we observed similar and supportive effects of ROCK inhibition on TGF-*β*3-induced Smad 2/3 activation and upregulation of the tendon marker scleraxis in both species. Furthermore, at least in human MSC, the fold change in scleraxis expression upon TGF-*β*3 and Y-27632 stimulation compared to the unstimulated control was much higher in the scaffold than in the monolayer culture. This might be due to stronger basal ROCK activation in the scaffold environment, entailing a stronger effect of its inhibition, yet this was not quantified in the current study. In this context, it should also be noted that ROCK inhibition alone did not reverse the higher scleraxis expression in scaffold-cultured human MSC. Thus, contrary to previous studies with artificial scaffolds [21, 33], the tenogenic stimulus provided by the native tendon scaffold appeared to be independent of Rho/ROCK signalling. Overall, our results imply that Rho/ROCK activity does not promote but rather counteract TGF-*β*3-induced tenogenic differentiation.

While this appears surprising against the background of previous studies on tenogenic signalling, our findings are in agreement with others who observed reinforcement of the TGF-*β* pathway with increased Smad 3 phosphorylation when using Y-27632 for ROCK inhibition [38]. There are different plausible explanations for these results. First, cellular tension, which is regulated by Rho/ROCK, regulates the localization and multimerization of the TGF-*β* receptor components. Specifically, ROCK inhibition was demonstrated to drive the receptor multimerization required for TGF-*β* downstream effects [39], which could be responsible for the reinforced TGF-*β*3 effects observed with ROCK inhibition in the current study. Secondly, ROCK is known to directly interact with the Smad 2/3 molecules and capable of phosphorylating the inhibitory linker region of these Smads, thereby inhibiting Smad activity [16, 40]. Thus, again the Smads 2 and 3 appear as possible key molecules in the crosstalk between ECM and TGF-*β* downstream signalling, with their activation or inhibition occurring through phosphorylation at different molecular sites. Finally, with regard to the tenogenic effects of the tendon ECM which appeared to be independent of Rho/ROCK, direct interactions between integrins and TGF-*β* receptors [41] as well as with the ECM, TGF-*β*, and its binding proteins [42] need to be considered.

We also observed some inconsistencies between human and equine MSC. In human monolayer MSC, qualitative evaluation of the cytoskeleton suggested that ROCK inhibition had already decreased until day 3, with possible implications for the quantitative day 3 results in these cells. Equine MSC showed overall less scleraxis upregulation, and in contrast to human MSC, the tendon scaffold did not increase but reduce the expression of all investigated tendon markers. Furthermore, the compensation and feedback mechanisms of human and equine MSC appeared to be different. While both species downregulated Smad 3 in the presence of TGF-*β*3, human MSC upregulated Smad 8 in turn, suggesting activation of this alternative pathway, and equine MSC upregulated TGF-*β*3, suggesting activation of a positive feedback loop. In addition, there were species differences in ECM gene expression. Most remarkably in the context of the current study, equine MSC but not human MSC downregulated collagen 1A2 when TGF-*β*3 stimulation was combined with ROCK inhibition.

While providing the first evidence for an inhibitory role of ROCK in tenogenic Smad 2/3 activation, the current study is limited by the number of donors included and the lack of further quantitative analysis on protein level; thus, it cannot clarify all questions it raised. Further studies are necessary to confirm our findings, to unveil more details of the intracellular signalling crosstalk, and to understand the observed species differences. Last not least, experiments with a longer follow-up are required to investigate if tenogenic differentiation would mature with or without continuous ROCK inhibition.

## 5. Conclusions

The current results support the hypothesis that Rho/ROCK inhibition promotes the TGF-*β*3/Smad 2/3 pathway in the context of tendon regeneration, while tenogenic effects of native tendon ECM are preserved. These findings provide new insight into a possible regulatory mechanism affecting tenogenic differentiation and make the Rho/ROCK pathway an interesting therapeutic target. Given that future studies will deliver promising long-term results and unveil more details on this mechanism, this could be useful to improve the therapeutic use of MSC, as ROCK-targeting priming regimes might promote overall tenogenic differentiation *in vivo* and prevent MSC maladaptations to pathologically altered ECM.

## Figures and Tables

**Table 1:** Human primers for real-time PCR.

Gene	Primer sequence	Accession	PCR product (bp)
GAPDH	For: CCACTCCTCCACCTTTGACG Rev: CCCTGTTGCTGTAGCCAAATTC	NM_001256799.1	101
Collagen 1A2	For: CAAGGACAAGAAACACGTCTGG Rev: GTTGGGTAGCCATTTCCTTGG	NM_000089.3	101
Collagen 3A1	For: TGAATATCGAACACGCAAGGC Rev: AAAGCAAACAGGGCCAACG	NM_000090.3	109
Scleraxis	For: GCGACGGCGAGAACACC Rev: TCCTTGCTCAACTTTCTCTGGTT	NM_001080514.1	73
Tenascin-C	For: CTCTGGAAGACACCGTTGGC Rev: GAAGTGGTGTTTCTTGGAAGCTG	NM_002160.3	101
Smad 3	For: GGTCTGCGTGAATCCCTACC Rev: TTATGTGCTGGGGACATCGG	NM_005902.3	294
Smad 8	For: CCTGTAGATGCCACAGCTGATA Rev: GGGAGGAAGCCTGGAATGTC	NM_001127217.2	151
TGF-*β*3	For: AGCGCTATATCGGTGGCAAG Rev: TCATCCTCATTGTCCACGCC	NM_003239.4	226

**Table 2:** Equine primers for real-time PCR.

Gene	Primer sequence	Accession	PCR product (bp)
GAPDH	For: TGGAGAAAGCTGCCAAATACG Rev: GGCCTTTCTCCTTCTCTTGC	NM_001163856.1	309
Collagen 1A2	For: CAACCGGAGATAGAGGACCA Rev: CAGGTCCTTGGAAACCTTGA	XM_001492939.1	243
Collagen 3A1	For: AGGGGACCTGGTTACTGCTT Rev: TCTCTGGGTTGGGACAGTCT	XM_001917620.2	216
Scleraxis	For: TACCTGGGTTTTCTTCTGGTCACT Rev: TATCAAAGACACAAGATGCCAGC	NM_001105150.1	51
Tenascin-C	For: TGAATATGGAATTGGAGTGTCTGC Rev: GGAGTTCTCCACACCAGAGTC	XM_023628743.1	147
Smad 3	For: GCTGTGTGAGTTCGCCTTCA Rev: CGGGGATGGAATGGCTGTAG	XM_023654980.1	164
Smad 8	For: AGCCTCCGTGCTCTGCATT Rev: CCCAACTCGGTTGTTTAGTTCAT	AB106117.1	200
TGF-*β* 3	For: TGCCCCAAAGGAATCACCTC Rev: GCGTTGTTTGGCGATATGCT	XM_001492687.6	89

## Data Availability

The raw data supporting the conclusions of this article will be made available by the authors, without undue reservation.

## Supplementary Materials

Supplementary Materials Supplementary file 1: human MSC characterization. Upper panel: representative images of Oil Red O staining (left) and von Kossa staining (right), showing adipogenic or osteogenic differentiation, respectively. Lower panel: representative histograms for surface antigen expression analysed by flow cytometry; antibody staining and analyses were performed as detailed in Schubert et al. (doi:10.1002/cyto.a.23240). The histograms display the relative number of events versus mean fluorescence intensity; blue represents Fixable E-Fluor® 780-stained MSC and red the MSC stained with the respective monoclonal antibody. For intracellular CD79*α* staining, an additional isotype control staining (yellow) was used to determine the percentage of positive cells.
