# Supplementary material for: Rho/ROCK Inhibition Promotes TGF-β3-Induced Tenogenic Differentiation in Mesenchymal Stromal Cells
Source: Stem Cells Int. 2021 Oct 8;2021:8284690. doi: 10.1155/2021/8284690 (PMC8519677; doi:10.1155/2021/8284690)

Supplementary file 1: Human MSC characterization

Upper panel:
Representative images of Oil Red O staining (left) and von Kossa staining (right), showing adipogenic or osteogenic differentiation, respectively.

Lower panel:
Representative histograms for surface antigen expression analyzed by flow cytometry; antibody stainings and analyses were performed as detailed in Schubert et al., 2018 (doi: 10.1002/cyto.a.23240). The histograms display the relative number of events versus mean fluorescence intensity; blue represents Fixable E-Fluor® 780-stained MSC, red the MSC stained with the respective monoclonal antibody. For intracellular CD79α staining, an additional isotype control staining (yellow) was used to determine the percentage of positive cells.

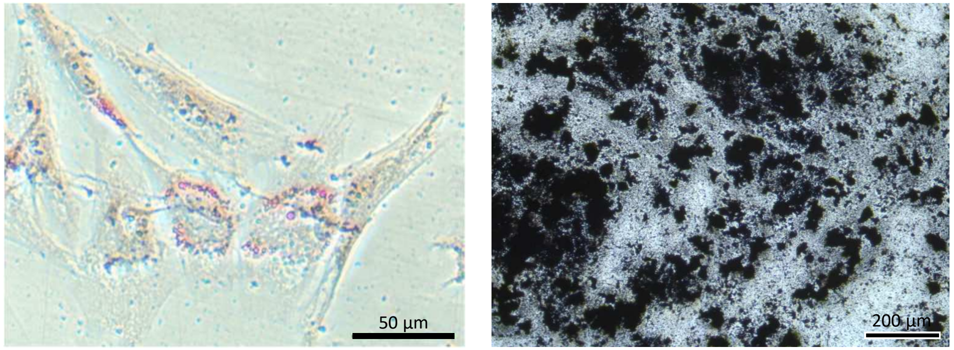

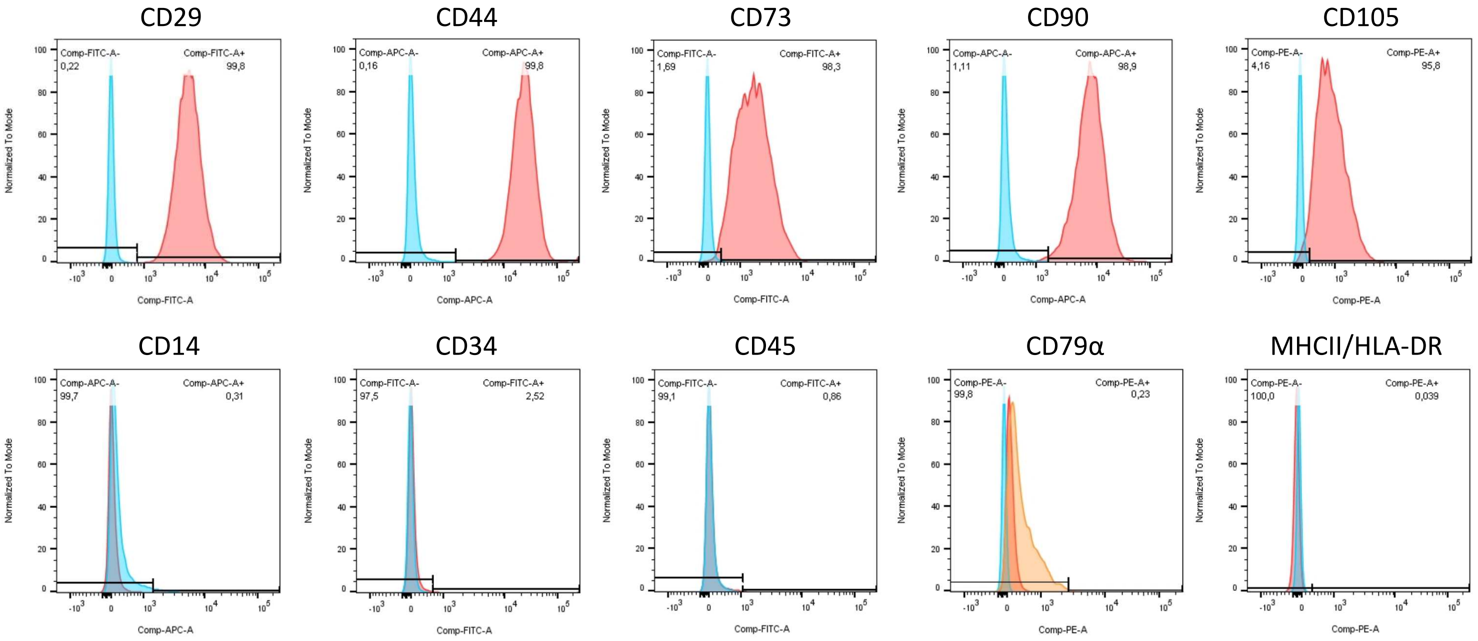

Supplement: Supplementary Materials — Supplementary file 1: human MSC characterization. Upper panel: representative images of Oil Red O staining (left) and von Kossa staining (right), showing adipogenic or osteogenic differentiation, respectively. Lower panel: representative histograms for surface antigen expression analysed by flow cytometry; antibody staining and analyses were performed as detailed in Schubert et al. (doi:10.1002/cyto.a.23240). The histograms display the relative number of events versus mean fluorescence intensity; blue represents Fixable E-Fluor® 780-stained MSC and red the MSC stained with the respective monoclonal antibody. For intracellular CD79α staining, an additional isotype control staining (yellow) was used to determine the percentage of positive cells. [file 8284690.f1.pdf]
